# *Escherichia coli* O157:H7 Converts Plant-Derived Choline to Glycine Betaine for Osmoprotection during Pre- and Post-harvest Colonization of Injured Lettuce Leaves

**DOI:** 10.3389/fmicb.2017.02436

**Published:** 2017-12-08

**Authors:** Russell A. Scott, Roger Thilmony, Leslie A. Harden, Yaguang Zhou, Maria T. Brandl

**Affiliations:** ^1^Produce Safety and Microbiology Research Unit, Agricultural Research Service, United States Department of Agriculture, Albany, CA, United States; ^2^Crop Improvement and Genetics Research Unit, Agricultural Research Service, United States Department of Agriculture, Albany, CA, United States

**Keywords:** foodborne pathogen, plants, produce, damage, osmotic stress response, compatible solute, osmoprotection

## Abstract

Plant injury is inherent to the production and processing of fruit and vegetables. The opportunistic colonization of damaged plant tissue by human enteric pathogens may contribute to the occurrence of outbreaks of foodborne illness linked to produce. *Escherichia coli* O157:H7 (EcO157) responds to physicochemical stresses in cut lettuce and lettuce lysates by upregulation of several stress response pathways. We investigated the tolerance of EcO157 to osmotic stress imposed by the leakage of osmolytes from injured lettuce leaf tissue. LC-MS analysis of bacterial osmoprotectants in lettuce leaf lysates and wound washes indicated an abundant natural pool of choline, but sparse quantities of glycine betaine and proline. Glycine betaine was a more effective osmoprotectant than choline in EcO157 under osmotic stress conditions *in vitro*. An EcO157 mutant with a deletion of the *betTIBA* genes, which are required for biosynthesis of glycine betaine from imported choline, achieved population sizes twofold lower than those of the parental strain (*P* < 0.05) over the first hour of colonization of cut lettuce in modified atmosphere packaging (MAP). The cell concentrations of the *betTIBA* mutant also were 12-fold lower than those of the parental strain (*P* < 0.01) when grown in hypertonic lettuce lysate, indicating that lettuce leaf cellular contents provide choline for osmoprotection of EcO157. To demonstrate the utilization of available choline by EcO157 for osmoadaptation in injured leaf tissue, deuterated (D-9) choline was introduced to wound sites in MAP lettuce; LC-MS analysis revealed the conversion of D9-choline to D-9 glycine betaine in the parental strain, but no significant amounts were observed in the *betTIBA* mutant. The EcO157 Δ*betTIBA*-Δ*otsBA* double mutant, which is additionally deficient in *de novo* synthesis of the compatible solute trehalose, was significantly less fit than the parental strain after their co-inoculation onto injured lettuce leaves and MAP cut lettuce. However, its competitive fitness followed a different time-dependent trend in MAP lettuce, likely due to differences in O_2_ content, which modulates *betTIBA* expression. Our study demonstrates that damaged lettuce leaf tissue does not merely supply EcO157 with substrates for proliferation, but also provides the pathogen with choline for its survival to osmotic stress experienced at the site of injury.

## Introduction

*Escherichia coli* serovar O157:H7 (EcO157) is a prevalent foodborne pathogen that has caused numerous outbreaks of human infection linked to the consumption of lettuce in the United States, Europe, and other industrialized countries (Franz and van Bruggen, 2008; Lynch et al., 2009; Mandrell, 2009; Pennington, 2010). While field studies have demonstrated the ability of EcO157 to persist on lettuce plants for a few days after inoculation or up to harvest, none have demonstrated an overall increase in the population size of the pathogen on lettuce leaves under field conditions (Islam et al., 2004; Barker-Reid et al., 2009; Fonseca et al., 2011; Moyne et al., 2011; Bezanson et al., 2012; Oliveira et al., 2012). Given the occurrence of outbreaks associated with lettuce, it is reasonable to hypothesize that EcO157 must encounter microsites that allow for its multiplication to an infectious dose on/in the plant tissue during the production and processing of lettuce.

We have reported previously that plant lesions promoted high multiplication rates of EcO157 on lettuce leaves (Brandl, 2008), and field studies by others showed that EcO157 (Aruscavage et al., 2008) and non-pathogenic *E. coli* (Barker-Reid et al., 2009; Harapas et al., 2010) survived at greater rates on mechanically injured than intact lettuce. Additionally, we have demonstrated previously that downy mildew infection sites on lettuce supported greater multiplication and survival rates of EcO157 than healthy leaf tissue under wet and dry conditions in the phyllosphere, respectively (Simko et al., 2015). Tip burn lesions, caused by a physiological disease of lettuce also hosted high densities of EcO157 (Brandl, 2008). In lettuce microbiome studies, infection with *Rhizoctonia solani* enriched for the presence of members of the Enterobacteriaceae in the phyllosphere, including a significant upshift in abundance of *Enterobacter* spp. within that community (Erlacher et al., 2014, 2015). Hence, compromised lettuce leaf tissue resulting from various insults provides new favorable niches for colonization and persistence of enteric pathogens and related species.

Although human pathogens may benefit from the release of substrates from damaged cells in plant lesions (Aruscavage et al., 2010; Kyle et al., 2010; Goudeau et al., 2013; George et al., 2016), physicochemical conditions resulting from the injury *per se* and from the plant defense response to wounding or infection may also contribute to the outcome of a contamination event at the site of injury. Our transcriptomic studies previously revealed that in addition to responding to oxidative and antimicrobial stress in romaine lettuce leaf lysate and in cut leaf tissue, EcO157 mounted a response to osmotic stress by upregulation of the *betA* and *betB* genes for GB synthesis while the *otsBA* genes for production of the osmoprotectant trehalose were strongly downregulated (Kyle et al., 2010). RNAseq data from an ongoing study in our laboratory corroborated these findings by revealing also an increased expression of the *betTIBA* genes and concomitant repression of *otsBA* in EcO157 during colonization of shredded Iceberg lettuce compared with that of intact lettuce (unpublished data). Correlations between the osmotic stress tolerance of human pathogens and their ability to colonize their animal hosts have been documented (Kunin et al., 1992; Wargo, 2013a). However, the role of the osmotic stress response in the survival and proliferation of human foodborne pathogens in the plant habitat has not been investigated to date and most of our understanding of the osmoprotectants used by EcO157 in the environment outside of its primary hosts comes from soil studies (Burgess et al., 2016).

Bacteria may scavenge osmoprotectants, such as GB and proline directly from their environment, whereas others are synthesized *de novo* (trehalose) or from environmental precursors, as is the case for choline uptake and its conversion to GB (Kempf and Bremer, 1998). The transport of choline and GB in *E. coli* is regulated by osmotic stress (Lamark et al., 1996) and mediated by BetT, ProP, and ProU transmembrane transporters (Ly et al., 2004). Once imported into the cell, choline may be converted by the choline dehydrogenase BetA and the betaine aldehyde dehydrogenase BetB to produce GB, which confers a high level of osmotolerance in *E. coli* (Landfald and Strøm, 1986). In response to osmotic stress, *E. coli* accumulates GB to a range of intracellular levels proportionally to the environmental water potential (Perroud and Le Rudulier, 1985; Larsen et al., 1987; Wood et al., 2001). While *otsBA* also is positively regulated by osmotic stress (via RpoS), the *otsA* gene product is suppressed post-translationally by high intracellular levels of GB (Giaever et al., 1988).

Choline is commonly found in plants, where it is formed in the cytosol and is incorporated into membranes as the polar head group of phosphatidyl choline (Summers and Weretilnyk, 1993), a major phospholipid component of plant membranes (Whitman and Travis, 1985; Vu et al., 2014). Among other quaternary compounds, choline may serve for production of compatible osmolytes to facilitate plant adaptation to saline and drought conditions (Rhodes and Hanson, 1993). Plants suffering from damaged tissue also face desiccation as water is lost from plant cells, therefore necessitating repair (Boerjan et al., 2003). Upon plant injury, chemical wound signals are produced by enzymatic reactions with membrane phospholipids; phosphatidic acid is a jasmonic acid precursor and itself a prominent signaling molecule generated when wounding triggers the membrane localization and activation of various phospholipases (Wang et al., 2000).

Given our previous observations of the increased expression of *bet* genes in EcO157 in two experimental model systems for mechanically injured lettuce leaves, we hypothesized that wounded lettuce leaf tissue inflicts hyperosmotic stress on EcO157 and that choline is likely available to the pathogen in plant wounds to serve as a precursor to the BetTIBA pathway. We therefore assessed the presence of the precursor choline and end product GB in both the bacterium and plant host wounds by quantification of these compounds using LC-MS analysis and an enzymatic assay. Furthermore, we determined the osmotolerance of a single BetTIBA mutant and a double BetTIBA-OtsBA mutant *in vitro* as well as their competitive fitness in various models of injured plant tissue.

## Materials and Methods

### Bacterial Strains and Culture Conditions

All strains and plasmids used in this study are listed in **Table 1**. *E. coli* O157:H7 strain TW14588, a clinical isolate from a 2006 outbreak attributed to Iceberg lettuce served at Taco John (Manning et al., 2008), was kindly provided by the Thomas Whittam STEC Center, MI and used for our studies. The genome sequence of this strain is available in GenBank (Acc. No. ABKY02000001- ABKY02000010). Unless mentioned otherwise, all strains were cultured in Luria-Bertani half-salt (5 g NaCl/L) (LBHS) broth and incubated at 28°C on a roller drum. LBHS broth was amended as appropriate with kanamycin (Km) (30 μg/ml), gentamicin (Gm) (15 μg/ml), chloramphenicol (Cm) (20 μg/ml), or carbenicillin (Cb) (50 μg/ml).

**Table 1:** Strains and plasmids used in this study.

	Relevant features	Source or reference
**Strains**		

***E. coli* O157:H7**		
TW14588	“Taco John” outbreak clinical strain from the Thomas Whittam STEC Center; genome-sequenced strain	Manning et al., 2008
TW14588::*aacC1*	TW14588 *att*Tn*7*::*aacC1*, Gm^r^	This study
TW14588::*cat*	TW14588 *att*Tn*7*::*cat*, Cm^r^	This study
TW14588::*cat*/pGT-Kan	TW14588::*cat* with pGT-KAN, Cm^r^, Gm^r^	This study
TW14588::*cat* /pMBotsBA	TW14588::*cat* with pBBR1-MCS-5-*otsBA*, Cm^r^, Gm^r^	This study
TW14588::*cat*/pMBbetTIBA	TW14588::*cat* with pBBR1-MCS5-*betTIBA*, Cm^r^, Gm^r^	This study
TW14588 Δ*betTIBA*	TW14588 Δ*betTIBA*::*cat*, Cm^r^	This study
TW14588 Δ*otsBA*	TW14588 Δ*otsBA*::*kan*, Km^r^	This study
TW14588 Δ*otsBA* Δ*betTIBA*	TW14588 Δ*otsBA*::*kan* Δ*betTIBA*::*cat*, Km^r^ Cm^r^	This study
TW14588 Δ*otsBA* Δ*betTIBA*/pMBbetTIBA	TW14588 Δ*otsBA*::*kan* Δ*betTIBA*::*cat* with pMBbetTIBA, Km^r^, Cm^r^, Gm^r^	This study
TW14588 Δ*otsBA* Δ*betTIBA*/pMBotsBA	TW14588 Δ*otsBA*::*kan* Δ*betTIBA*::*cat* with pMBotsBA, Km^r^, Cm^r^, Gm^r^	This study
TW14588 Δ*otsBA*/pMBotsBA	TW14588 Δ*otsBA*::*kan* with pMBOtsBA, Km^r^, Gm^r^	This study
TW14588 Δ*betTIBA*/pMBbetTIBA	TW14588 Δ*betTIBA*::*cat* with pMBBetTIBA, Cm^r^, Gm^r^	This study
***Pseudomonas* spp.**		
*Pseudomonas aeruginosa* PAO1	Wild type *P. aeruginosa*	Holloway, 1955
*Pseudomonas syringae* pv. syringae B728A	Wild type *P. syringae*	Loper, 1987
*Pseudomonas syringae* pv. tomato DC3000	Wild type *P. syringae*	Cuppels, 1986

**Plasmids**		

pSTNSK	pST76-K::*tnsABCD*; Km^r^	Crépin et al., 2012a
pGP-Tn*7*-Gm	pGP704::Tn*7-aacC1*; Cb^r^ Cm^r^	Crépin et al., 2012a
pGP-Tn*7*-Cm	pGP-Tn*7*-FRT::*cat*; Cb^r^ Cm^r^	Crépin et al., 2012b
pBBR1MCS-5	Broad host range cloning vector, Gm^r^	Kovach et al., 1995
pMBotsBA	pBBR1MCS-5 with *otsBA* from EcOI157 TW14588 cloned into SpeI-SacI; Gm^r^	This study
pMBbetTIBA	pBBR1MCS-5 with *betTIBA* from EcO157 TW14588 cloned into SpeI-SacI; Gm^r^	This study
pGT-Kan	pPROBE-GT with P_kan_-*gfp;* Gm^r^	Brandl et al., 2005


*Km^*r*^, kanamycin resistant; Cm^*r*^, chloramphenicol resistant; Gm^*r*^, gentamicin resistant, Cb^*r*^, carbenicillin resistant.*

Lettuce leaf lysate was used immediately after preparation, and when appropriate, was amended with a NaCl solution to a final concentration of 550-, 650-, and 750 mM, or with DDI H_2_O at 10% volume in the control. The leaf lysate then was dispensed at 500 μl per well into 48-well plates prior to inoculation at 2 × 10^7^ cells/ml. The cultures in lysate were incubated at 28°C with constant shaking in a BIOTEK Epoch II plate incubator and reader. Inoculum suspensions were prepared from overnight cultures in the stationary phase of growth. Cells were centrifuged, washed twice in 10 mM KPO_4_ (KP) buffer (pH 7), and resuspended in 1 mM KP buffer at the desired cell concentration based on OD_600_.

### Strain Construction

The EcO157 TW14588 WT strain was marked with a gentamicin- or chloramphenicol resistance cassette introduced on the chromosome at the *att*Tn*7* site using the mini-Tn*7* procedure described by Crépin et al. (2012a) with minor modifications. Plasmids were kindly provided by Dr. Charles Dozois (INRS-Institut Armand-Frappier, Laval, QC, Canada). The helper plasmid pSTNSK was introduced into the WT strain by electroporation, which was transformed again with pGP-Tn*7*-Gm or pGP-Tn*7*-Cm to generate TW14588::*aacC1* and TW14588::*cat*, respectively. The transformants were selected on LBHS agar with the appropriate antibiotic and incubation at 37°C. Colonies were restreaked also onto LBHS containing carbenicillin to confirm loss of pSTNSK, and insertion of the cassette at the *att*Tn*7* site was verified by PCR.

Deletion mutants were generated via lambda Red-mediated recombination using pKD4 or pKD3 (Datsenko and Wanner, 2000) and replacing *otsBA* or *betTIBA* with a kanamycin- and chloramphenicol resistance cassette, respectively. The double deletion mutant was constructed by deletion of *betTIBA* in the *otsBA* mutant, resulting in a strain marked with both kanamycin and chloramphenicol resistance. The deletion of *otsBA* and *betTIBA* was verified by PCR. PCR primers used for mutagenesis and verification of deleted target genes in the mutants are listed in Supplementary Table S1. The *otsBA* and *betTIBA* complementation plasmids were constructed by PCR cloning and ligation into SpeI and SacI restriction sites of pBBR1MCS-5 (Kovach et al., 1995) to generate pMBotsBA and pMBbetTIBA, respectively, using primers described in Supplementary Table S1. Complemented strains were generated by transformation of the mutants via electroporation.

### Plant Material and Growth Conditions

Iceberg Lettuce cv. Salinas seeds were kindly provided by Ivan Simko (USDA, Agricultural Research Service, Salinas, CA, United States). Plants were grown in Supersoil potting mix with approximately 150 mg of Osmocote Plus (15-9-12) at 22°C with a 14-h photoperiod for 6–8 weeks in a plant growth chamber (Percival Scientific, Inc.), and fertilized weekly with 20-20-20 (N-P-K) liquid fertilizer past the five expanded leaf-growth stage. For experiments with cut lettuce in modified atmosphere packaging (MAP) bags, mature Iceberg lettuce heads were acquired from local commercial sources, trimmed of outer leaves, and the leaves shredded into 2-mm wide strips with a sharp knife.

Lettuce leaf lysate was prepared as described previously with modifications (Kyle et al., 2010). Iceberg lettuce heads were obtained commercially and all portions except the outermost leaves and inner-most achlorophyllous heart were used. The leaves were homogenized in a Omega juicer (Omega Model No. 8003), with the homogenate kept on ice during production and immediately centrifuged at 7,000 rcf at 4°C for 10 min to pellet chloroplasts and plant debris. The supernatant was sterilized by passage through 0.45 μm- then through 0.2 μm pore-size filters.

### Plant Inoculations and Recovery of EcO157 from Wounds

For single inoculations of MAP lettuce, the WT strain (TW14588::*cat*) and the single *betTIBA* mutant (Δ*betTIBA*::*cat*) were inoculated individually onto the bagged cut leaves. In competitive fitness studies, the WT strain (TW14588::*aacC1*) was mixed with the double mutant Δ*otsBA*::*kan* Δ*betTIBA*::*cat* in a 1:1 ratio prior to co-inoculation.

For inoculation of cut (shredded) lettuce, square bags (24 cm × 24 cm) were made with MAP film commercially used for processed Iceberg lettuce and filled with 226 g lettuce, keeping bag size to product weight ratio as per commercial standards. Each bag received 2 ml inoculum at 1 × 10^5^ cells/ml that was distributed by gently shaking the bag. Bags were flushed three times with N_2_ gas, completely sealed and incubated at 24°C. Atmospheric O_2_ and CO_2_ contents in the bags were determined with a MOCON PAC CKECK O2/CO2 Analyzer (Model 650 EC). For bacterial cell recovery, the leaf material from each bag was transferred to Filtra-Bags (LabPlas, SCT07012A) and processed with 200 ml 10 mM KP buffer in a stomacher for 2 min. The resulting suspension was dilution-plated onto LBHS agar with appropriate antibiotics for CFU determinations of each strain.

For inoculation of leaf wounds on potted lettuce plants, 12″ straight dressing forceps with a 35 mm × 3 mm serrated surface area were immersed at the tip into inoculum suspensions of 1 × 10^7^ cells/ml in 1 mM KP buffer, twice blotted on Whatman #42 filter paper and used to generate one wound in the middle of the leaf blade and away from the midvein. This resulted in an initial inoculum level of approximately 5 × 10^4^ CFU/wound. Plants were incubated at 22–25°C and under 90–100% RH to promote microbial growth, but free water was absent macroscopically from the plant surface. Wounded tissue was sampled immediately after inoculation by wounding, and thereafter at indicated times, by cutting out a 7 mm diameter disk spanning initial wound striations. The disks were homogenized in 1 ml 10 mM KP buffer with 12 glass beads (Fisher Scientific, 11-312A), at 4°C for 2 min in a Mini-Bead beater 96+ 1HP (11.2A @115 VAC) (Biospec products). Five and ten disks were sampled at random from replicate leaves and replicate plants at the time of inoculation, and at each sampling time thereafter, respectively. The disk homogenate was dilution-plated onto LBHS agar containing appropriate antibiotics for CFU determinations of each strain.

### Compatible Solute Quantification by LC-MS

For analysis of uninoculated lettuce lysates, lysates were sampled by mixing 1:1 (v:v) with ice cold HPLC-grade 50% MeOH, centrifugation at 15,000 RCF and 4°C, and 500 μl supernatant dried in a SpeedVac, reconstituted in a smaller volume prior to filtration using 10K molecular weight cutoff microcentrifuge columns, and used for LC-MS. For quantification of osmoprotectants in EcO157 cells cultured in lettuce lysate, bacterial cells were recovered from the lysate by centrifugation and the pellets washed twice with ice-cold 1 M NaCl to prevent spontaneous expulsion/export of intracellular osmoprotectants, then frozen at -20°C prior to lysis (Fagerquist et al., 2014) with zirconia silica beads in 300 μl Nuclease-Free H_2_O by bead beating as described above. Cell lysates were centrifuged and the supernatant transferred to MeOH 1:1, then centrifuged again and the supernatant used for mass spectrometry. Choline, glycine betaine, and proline present in lettuce lysates and cells grown in lettuce lysates were analyzed by injection of samples into a nanoflow Reprocsil-PUR C18-AQ column (New Objective, Woburn, MA, United States) with a Thermo EZ-nano HPLC followed by detection on a Thermo Orbitrap Elite ion mass spectrometer (Thermo Fisher, Waltham, MA, United States). The Orbitrap was operated in positive ion mode, with the mass resolution set to 30,000. MS data were collected from 95 to 200 m/z. Analytes were minimally retained and eluted with isocratic flow (400 nL/min) of 2% MeCN in HOH with 0.1%formic acid (Optima LC-MS grade, Fisher Scientific).

For D9 tracing experiments, EcO157 cells were inoculated onto MAP lettuce with a 1 ml suspension of 1 × 10^9^ cells/ml containing [1 M] D9-choline, and incubated as described above, with the exception of non-MAP bags sealed using ambient atmosphere, and uninoculated bags which were MAP-sealed but received no inoculum or D9-choline. At 6 h post-inoculation, MAP, non-MAP, and uninoculated lettuce samples were processed by washing with 50 ml 1 M ice cold NaCl to recover cells, dilute away extracellular osmoprotectants, and retain intracellular osmoprotectants, and a portion retained for dilution plating. Cells were then centrifuged at 26,640 *g*, and lysed as described above for EcO157 cultured in lettuce lysates. Quantification was performed by comparison of relative peak intensity of the monoisotopic molecular ions in spectra generated by averaging scans across the analytes elution off the column with D9-choline and D11-betaine (Cambridge Isotope Laboratories, Tewksbury, MA, United States) co-injected as internal standards.

Analysis of D9-GB in the lysates was carried out on a Thermo Orbitrap Elite ion mass spectrometer fitted with an Agilent 1100 HPLC. A Thermo HESI source was used with the electrospray voltage set to 4 kV, sheath gas set to 10, auxiliary gas at 5, and capillary inlet temperature at 295°C. The MS was operated at 15000 resolution and mass spectra were collected in SIM mode monitoring five mass windows: 117.6–118.6, 128.7–129.7, 103.6–104.6, and 112.7–113.7 Da, and 126.6–127.6 for D9-Choline, D11-GB, native Choline, native GB, and D9-GB, respectively. Data were processed with Thermo Xcalibur Quant Browser using Genesis peak integration with 15 smoothing points.

Normal phase chromatography prior to MS analysis was carried out with a 15 cm × 2.1 mm Diamond Hydride column coupled with a 70000 HG5 Diamond Hydride guard column (MicroSolve, Leland, NC, 28541). The HPLC was operated at 400 μL/min with Fisher Optima LC-MS grade solvents (Fisher Scientific). The gradient of A (water with 0.1% formic acid) and solvent B (acetonitrile with 0.1% formic acid) was used as follows: 92% B for 1 min, to 20%B at time 15 min, hold for 3 min, then return to 92%B at time 20 min. The divert valve was programmed to divert to waste from time 0 to 4 min, to the source from 4 to 14 min, and to waste from 14 to 20 min. Prior to injection, samples were mixed 1:1 with D9-Choline and D9-Betaine (Cambridge Isotopes, Tewksbury, MA 01876, United States) to a final concentration of 20 pM/μL deuterated standards with the exception of runs designed to detect D9-GB from bacterial cell conversion of D9-choline in inoculated MAP lettuce, in which case only D11-GB was added to the samples in the same manner as D-9 Choline had been added previously for those experiments.

### Choline Quantification by Enzyme Assay

For quantification of choline in MAP cut lettuce, leaves were processed and bagged as described above. Immediately after bagging the cut leaves, and at various time after incubation in MAP, 20 ml of ice-cold H_2_O was used to wash off choline from the lettuce pieces by gently moving the H_2_O over the plant material in the bag. The resulting wash water was then mixed 1:1 with ice cold HPLC-grade MeOH and stored at -20°C. Choline was quantified with a choline assay kit (BioVision, Inc.) according to the manufacturer’s instructions with modifications. Samples in MeOH:H_2_O were dried in wells of 96-well plates in a SpeedVac and reconstituted in 20 μL choline assay buffer. Diluted choline probe was added rapidly to reagents in control wells and the plate read in a BioTek plate reader (exc. 530 nm, emm. 590 nm) to establish background. Diluted enzyme mix was then immediately added to each well. The reactions were monitored at 28°C until OD_590_ reached a maximum, and absorbance data from test samples were interpreted relative to internal choline standards (1 to 100 pmoles reaction^-1^).

### Microscopy

For visualization of the pathogen in inoculated leaf wounds, EcO157 TW14588 was transformed with pGT-Kan (Brandl et al., 2005), which is stably maintained in *E. coli* and allows for the constitutive expression of the green florescent protein (GFP) gene. Leaves of young lettuce plants were wound-inoculated with forceps as described above, and the plants incubated at 28°C and 90–100% RH. Small disks were sampled from the wounded tissue immediately and 6 h after wounding/inoculation. The GFP-labeled bacteria on/in the tissue were visualized with a Leica confocal microscope TCS SP5 (Leica Microsystems, Germany) and pseudo-3D images were constructed by projected series of multiple optical scans in the *z* plane.

### Statistical Analysis

Statistical analysis of the data was performed with GraphPad Prism version 7.03 (GraphPad Software, San Diego, CA, United States^1^). Direct comparison of the log-transformed population size of an EcO157 mutant with that of the WT strain was done with the unpaired Student’s *t*-test when appropriate. Percentage values obtained for representation of the mutant strain in the total EcO157 population in competitive fitness experiments were transformed to arcsin(squareroot %) before analysis. One-way ANOVA was used for comparison of more than two groups, followed by Tukey’s or Dunnett’s multiple comparison test. Differences were considered significant at *P* < 0.05.

## Results

### Contributions of Osmoprotectants and Osmotic Stress Resistance Genes in Hypertonic Minimal Medium

**FIGURE 1:**
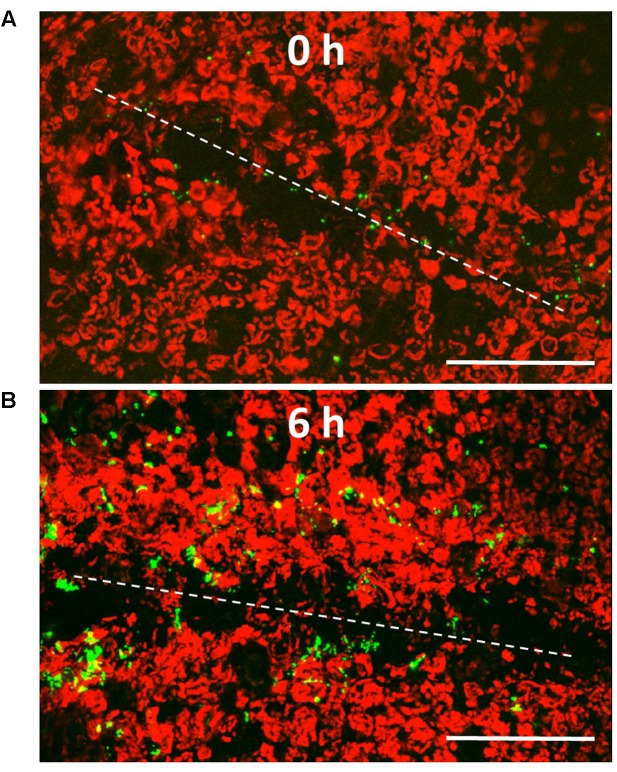
Pseudo-3D confocal scanning laser microscopy images of GFP-EcO157 in wounded lettuce leaf tissue. Green fluorescent EcO157 cells in the leaf wounds **(A)** immediately after inoculation by wounding (0 h), and **(B)** 6 h after inoculation and wounding. White dotted lines delineate the wound generated by a single forcep striation. The red signal originates from the autofluorescence of the chloroplasts present in the leaf cells. Scale bars: 100 μm.

**FIGURE 2:**
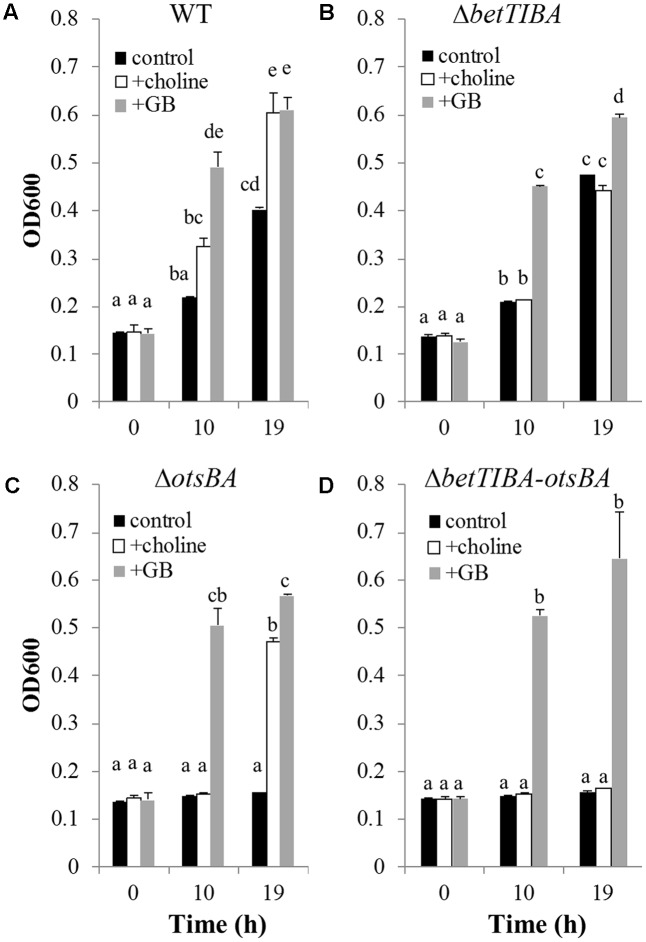
Chemical complementation of EcO157 WT strain and osmotic solute accumulation mutants. Growth of **(A)** WT, **(B)** *betTIBA*-, **(C)** *otsBA*-, and **(D)** *betTIBA-otsBA* mutants in M9-glucose minimal medium amended with 500 mM NaCl, and with 1 mM choline (+choline), or 1 mM glycine betaine (+GB), or an equivalent volume of H_2_O (control). Each bar represents the mean of determinations from two replicate cultures in wells of a 48-well plate, and standard error of the mean. Within each panel, bars marked with a same letter indicate that means are not significantly different based on Tukey’s multiple comparison test (*P* > 0.05).

Lettuce leaf wound colonization by EcO157 TW14588 occurred rapidly at temperatures conducive to multiplication of the pathogen. As illustrated in **Figure 1**, the population densities of GFP-labeled TW14588 evolved from single cells at distant locations to cell assemblages along the site of injury only 6 h after inoculation of the leaves of potted lettuce plants by wounding the leaf blade with contaminated tweezers. Given that the *betTIBA* genes were induced in lettuce leaf lysates (Kyle et al., 2010), and in cut vs. intact lettuce under MAP conditions (unpublished data), we hypothesized that the presence of choline, the precursor of the BetTIBA pathway, could convey a growth advantage to EcO157 under osmotic stress conditions *in planta*. We first investigated the role of this pathway under osmotic stress conditions *in vitro* and observed that the growth of EcO157 in M9-glucose minimal medium amended with 500 mM NaCl was enhanced by amendment with choline, as well as with the pathway end-product GB (**Figure 2A**).

A marked site-directed deletion mutant of the *betTIBA* region was constructed by the λ Red recombinase approach (Datsenko and Wanner, 2000) (**Table 1**). The resulting strain MB1141 (TW14588Δ*betTIBA*::*cat*) exhibited growth similar to that of WT in M9 medium in the absence of osmotic stress (data not shown), as well as under osmotic stress in the absence of osmoprotectants (control) (**Figures 2A,B**). Consistent with the requirement of the *bet* genes for utilization of choline for osmoprotection, the *betTIBA* mutant did not receive a growth advantage under osmotic stress by addition of choline (**Figure 2B**). However, addition of GB restored growth of the *betTIBA* mutant to the same levels as those of the WT grown in the presence of choline or GB under these conditions (**Figures 2A,B**).

Because growth still occurred in the *betTIBA* mutant at high osmolyte concentrations (**Figure 2B**), the compensatory role of the *otsBA* operon in adaptation of EcO157 to osmotic stress was assessed in a WT and in a *betTIBA* mutant background. A mutant with a deletion in *otsBA* was constructed via λ Red recombinase by replacement of this operon with a kanamycin resistance cassette in the WT and in the *betTIBA* mutant background, resulting in the single mutant, strain MB962 (TW14588Δ*otsBA::kan)* and the double mutant, strain MB1145 (TW14588Δ*betTIBA*::*cat-*Δ*otsBA::kan*), respectively (**Table 1**). The WT, and *otsBA* and *betTIBA-otsBA* mutants displayed similar growth in M9 medium without additional osmoticum (data not shown), but the *otsBA* and *betTIBA-otsBA* mutants equally failed to grow in M9 medium containing 500 mM NaCl and lacking choline (**Figure 2**). Whereas the single *otsBA* mutant benefited from the presence of choline for growth under osmotic stress (**Figure 2C**), the double *betTIBA-otsBA* was not rescued by the addition of choline, although it displayed growth similar to that of the WT and both single *otsBA* and *betTIBA* mutants in the presence of GB (**Figure 2D**).

**FIGURE 3:**
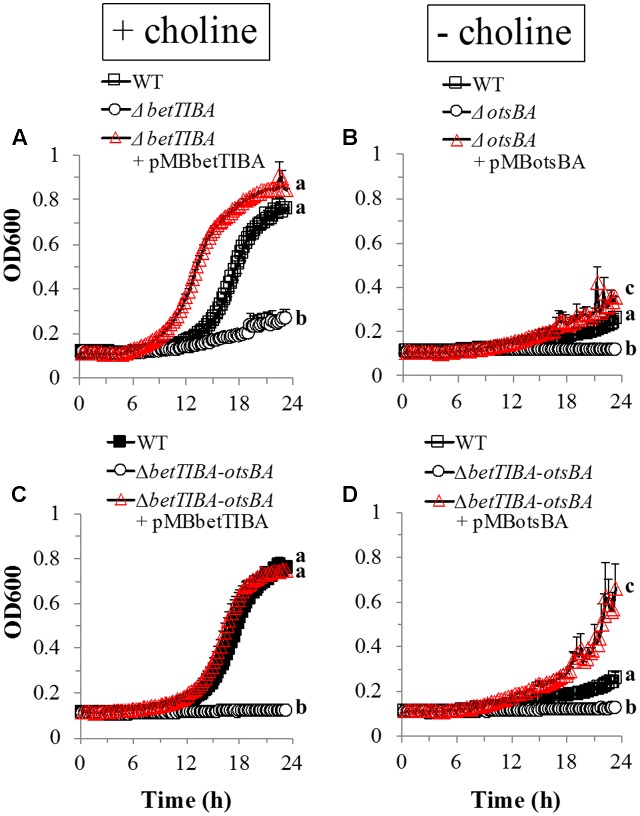
Genetic complementation of EcO157 mutants with a deletion in osmoprotectant accumulation operons. The *betTIBA*-, *otsBA*-, and *betTIBA-otsBA* mutants were complemented with **(A,C)** pMBbetTIBA or with **(B,D)** pMBotsBA. Strains were grown in M9-glucose minimal medium with 500 mM NaCl and with **(A,C)** or without **(B,D)** 1 mM choline. Each point represents the mean of determinations from two replicate cultures in wells of a 48-well plate, and standard error of the mean. Within each panel, data series marked with different letters indicate that the mean of the last three OD_600_ values in the time course for any given strain are significantly different based on Tukey’s multiple comparison test (*P* < 0.05).

Tolerance of the *betTIBA*, *otsBA*, and double *betTIBA-otsBA* mutants to osmotic stress was restored genetically by complementation with expression of the respective operons driven by their native promoter (**Figure 3**). When choline was added to hypertonic M9 medium, the presence of pMBbetTIBA in the *betTIBA* mutant and in the *betTIBA-otsBA* mutant enhanced their growth to at least that achieved by the WT strain (**Figures 3A,C**, respectively). When the *otsBA* and *betTIBA-otsBA* mutants were transformed with pMBotsBA, they displayed greater growth rates than the WT in hypertonic M9 medium without choline, which was not provided in order to remove the osmoprotective effect via BetTIBA (**Figures 3B,D**). All mutants and their complemented derivatives grew similarly to WT in M9 without osmotic stress (data not shown).

### MS Analysis of Osmoprotectants in EcO157 Cultured in Lettuce Lysate

**FIGURE 4:**
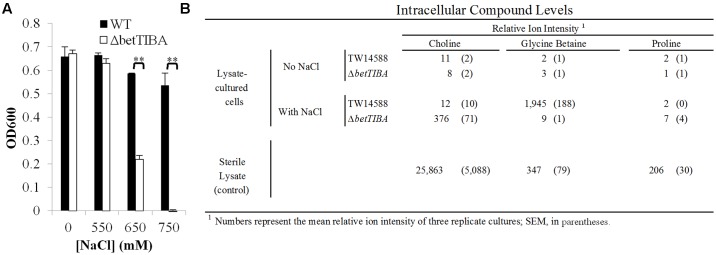
Growth and osmoprotectant accumulation in EcO157 WT and *betTIBA* mutant cells in lettuce lysates. **(A)** Growth after 24 h incubation in lettuce lysate amended with 550, 650, or 750 mM NaCl, or an equivalent volume of H_2_O (0 mM NaCl). Each point represents the mean of determinations from two replicate cultures in wells of a 48-well plate, and standard error of the mean. ^∗∗^ indicates significant difference by Student’s *t-*test (*P* < 0.01). **(B)** Relative intracellular levels of choline, glycine betaine, and proline in WT and *betTIBA* mutant cells recovered after 24-h growth in lettuce lysates containing 0 or 650 mM NaCl. Levels of same compounds in sterile lettuce lysates incubated under the same conditions are also shown. Numbers represent the mean relative ion intensity of three replicate cultures, with standard error of the mean provided in parentheses, as determined by MS analysis.

Lettuce leaf lysate first was used as a proxy for the chemical environment that EcO157 cells would experience when exposed to the contents of broken leaf cells, as we described previously (Kyle et al., 2010). Growth in lettuce lysate (amended with H_2_O at 10% final to adjust the volume in this control for addition of NaCl under hypertonic conditions) was similar in the WT and *betTIBA* single mutant, as well as in lysate with NaCl at 550 mM. However, upon addition of NaCl to 650 and 750 mM final concentration, the end-point absorbance of the *betTIBA* mutant cultures in lysate decreased sharply and was significantly different than that of the WT (Student’s *t*-test, *P* < 0.01) this was equivalent to a 2.25 and 12.14× difference in cell concentration (CFU/ml), respectively (**Figure 4A**).

To further test a model wherein choline is abundant in plant wounds and imported into EcO157 to be converted into GB, the relative intracellular abundance of these compounds was measured in WT and *betTIBA* mutant cells cultured in lettuce lysate using LC-MS analysis. As per relative ion intensity, both choline and GB accumulated in the control lysate (amended with H_2_O only) to lower levels than in lysate amended with 650 mM NaCl (**Figure 4B**). Under the latter hypertonic condition, the *betTIBA* mutant accumulated 31-fold greater quantities of the precursor choline than the WT, which in turn produced 216-fold more of the pathway end product GB than the mutant. Very low levels of proline, which can serve as an important osmoprotectant in *E. coli* (Kempf and Bremer, 1998), were detected in both strains even in the presence of high NaCl concentrations. Furthermore, quantities of this osmoprotectant were only 0.7% those of choline in the uninoculated sterile lettuce leaf lysate; GB levels were similarly low, at only 1.3% those of choline. Of note, while we observed that choline and GB serve in EcO157 TW14588 and EDL933 as osmoprotectants, these strains do not utilize choline or GB as sole carbon sources as certain *Pseudomonas* strains do (Chen and Beattie, 2008; Wargo et al., 2008) (Supplementary Figure S1).

### D9-Choline Tracing in EcO157 and Fitness of the *betTIBA* Mutant in Lettuce Wounds

**FIGURE 5:**
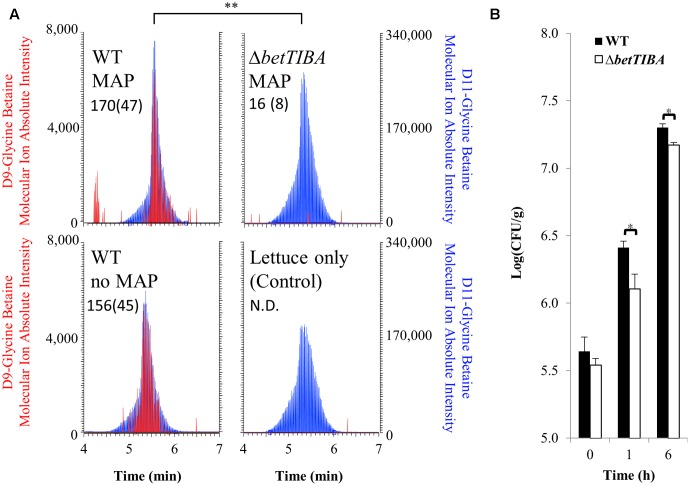
HPLC-MS quantification of D9-choline conversion to D9-GB in EcO157 WT and *betTIBA* mutant, and their growth in MAP cut lettuce. **(A)** Representative MS ion traces of D9-GB (left axis, red trace) from quantitative analysis in WT and *betTIBA* mutants cells recovered 6 h after single inoculation of their suspension amended with D9-choline onto cut lettuce incubated in bags under MAP (upper traces), or at room atmosphere (no MAP) (lower left trace). The lower right trace illustrates the lack of detection of D9-GB in uninoculated lettuce spiked with D9-choline and incubated for 6 h in MAP (control). The trace for D11-GB, which served as an internal standard is also shown (right axis, blue trace). Left and right axes indicate relative molecular ion intensity for representative peaks obtained for each treatment. Each trace is also shown with the amount of D9-GB produced by the strains; values are expressed as μmoles detected per L cell volume assessed in each bag (inferred from CFU determinations and assumption of 1 cubic μm volume per cell). Each value represents the mean from three replicate injections of samples from three replicate bags, with standard error of the mean provided in parenthesis. **(B)** Population dynamics of the WT and *betTIBA* mutant after their individual inoculation onto MAP cut lettuce. Each bar represents the mean population size per g cut lettuce from three replicate bags; error bars are standard error of the mean. ND, not detected. ^∗^ and ^∗∗^ indicate significant differences by Student’s *t-*test with *P* < 0.05 and *P* < 0.01, respectively.

In order to determine the activity of the BetTIBA pathway in EcO157 during colonization of injured lettuce leaves, deuterated choline (D9-choline) was fed to the TW14588 WT and *betTIBA* mutant by its addition to cell suspensions inoculated onto cut (shredded) lettuce. LC-MS analysis revealed that after 6 h of incubation under MAP conditions, D9-GB accumulated to 170 ± 47 μmoles L^-1^ cell volume in the WT whereas accumulation was minimal in the *betTIBA* mutant and 10.8-fold lower than in the WT cells (Student’s *t*-test, *P* < 0.01) (**Figure 5A**, upper panel). D9-GB levels in the WT in cut lettuce packaged under ambient atmosphere were 156 ± 45 μmoles L^-1^ cell volume and similar to those in MAP. D-9 GB was not detected in pellets recovered from uninoculated MAP cut lettuce (control) incubated for 6 h (**Figure 5A**, lower panel).

Given the above evidence that the BetTIBA pathway was active in TW14588 on injured leaf tissue, the WT and *betTIBA* mutant were compared for their colonization of cut lettuce leaves. Both strains multiplied rapidly on cut leaves under MAP conditions, but the population sizes of the WT strain were significantly greater than those of the mutant (Student’s *t*-test, *P* < 0.05) by 2.0- and 1.4-fold after 1- and 6 h incubation, respectively (**Figure 5B**).

### Comparative Growth of WT, Single *otsBA* Mutant and Double *betTIBA-otsBA* Mutant in Hypertonic Lettuce Lysate

**FIGURE 6:**
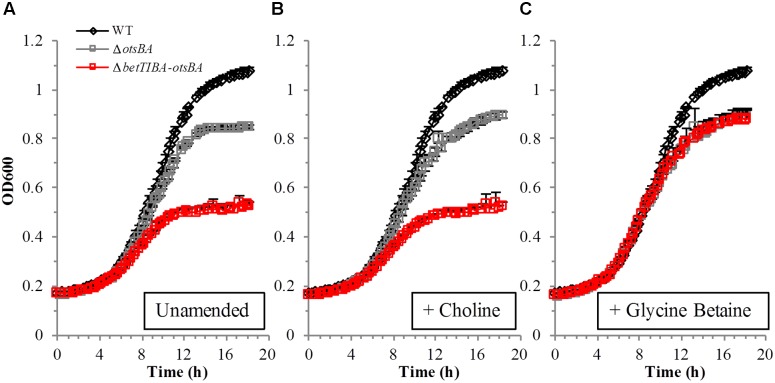
Growth of EcO157 WT, and *otsBA* and *betTIBA-otsBA* mutants in hypertonic lettuce lysates. Cells were cultured in lysates amended with 800 mM NaCl, and with **(A)** H_2_O (control), **(B)** 1 mM choline, and **(C)** 1 mM GB. Each point represents the mean of determinations from two replicate cultures in wells of a 48-well plate, and standard error of the mean.

With the knowledge that EcO157:H7 has several redundant pathways to respond to osmotic stress and that free GB and proline as available compatible solutes were detected in low amounts in plant cells (**Figure 4B**), a double mutant in which *de novo* trehalose synthesis could not serve a compensatory role in the absence of BetTIBA was investigated as well. The deletion of *otsBA* was important also because the product of the BetTIBA pathway, GB, is known to repress OtsBA in a post-translational manner (Giaever et al., 1988) and therefore, the deletion of *betTIBA* may have resulted in the re-activation of OtsBA in this single mutant. The *betTIBA-otsBA* double mutant was severely impaired in growth in lettuce lysate amended with NaCl, compared with the WT and with the single *otsBA* mutant (**Figure 6A**) despite that the latter likely benefited from choline availability and the activity of the BetTIBA pathway under these conditions, as shown in **Figures 4**, **5**. Addition of choline to the lettuce lysate did not increase the initial growth rate of the *otsBA* mutant, but promoted slightly greater cell concentrations in late exponential phase, likely because the natural pool of choline in the lysate provided osmoprotection in the early phase of growth (**Figure 6B**). However, addition of GB to the lysate strongly enhanced the growth of the double mutant since it curtailed the requirement of the BetTIBA pathway for use of GB as an osmoprotectant (**Figure 6C**). This observation suggests that although GB is present in significantly lower amounts in leaf lysate than choline (**Figure 4**), its uptake in damaged leaf tissue may offer some osmotic protection when the OtsBA and BetTIBA pathways are not present or active in EcO157.

### Competitive Fitness of a Double *betTIBA-otsBA* Mutant in Lettuce Wounds

**FIGURE 7:**
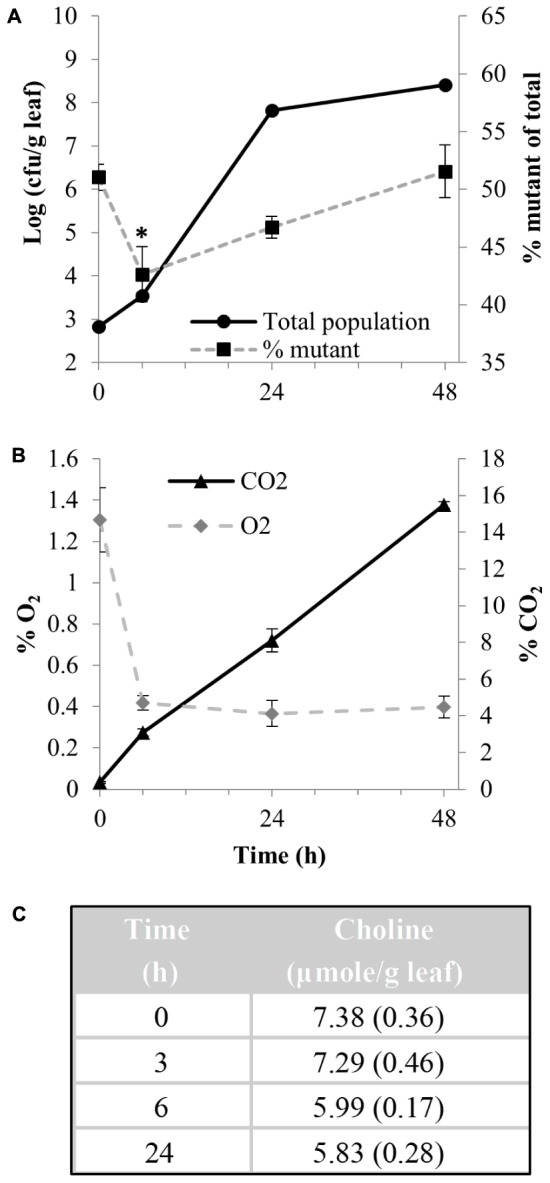
Competitive fitness of EcO157 WT and *betTIBA-otsBA* mutant in MAP cut lettuce, and chemical environment determinations. **(A)** Total EcO157 population size per g cut lettuce (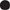) and percentage of the *betTIBA-otsBA* mutant in the total EcO157 population (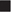) after their co-inoculation onto MAP cut lettuce. **(B)** Atmospheric O_2_ and CO_2_ content in the MAP bags from which the EcO157 strains were recovered. **(C)** Choline content in washes of MAP cut lettuce over time as measured via enzyme assay. All values represent mean measurements from three replicate bags. Standard error of the mean given as error bars or in parentheses. ^∗^ indicates a significant difference in the % of mutant over total EcO157 cells at 6 h compared with the initial % at the time of inoculation (0 h), based on Student’s *t*-test on transformed % data (*P* < 0.05).

Population dynamics following co-inoculation of the *betTIBA-otsBA* mutant and WT strains in a ratio of 1:1 onto shredded MAP lettuce showed that the total EcO157 population increased 5-log over 48 h, and that the proportion of the mutant decreased significantly from 51.1 to 42.6% over the first 6 h of incubation [arcsin(squareroot %)-transformed data; Student’s *t*-test, *P* < 0.05], but increased thereafter (**Figure 7A**). This trend in the competitive fitness of the mutant was concomitant with a sharp drop in O_2_ content from 1.3 to 0.4% in the bag atmosphere during the first 6 h to remain constant thereafter, and a steady increase in CO_2_ content throughout incubation due to plant respiration (**Figure 7B**). Choline was quantified in the washes of MAP shredded lettuce in order to assess its availability for uptake and transformation into GB in EcO157 cells in the injured tissue. Choline that leaked from the damaged tissue was abundant at the time of wounding the leaves by cutting (shredding) and decreased by 21% within 24 h after incubation under MAP conditions (**Figure 7C**).

**FIGURE 8:**
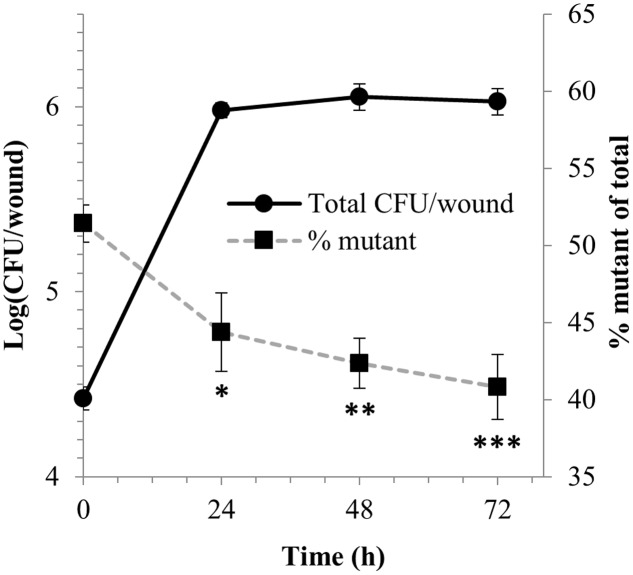
Competitive fitness of EcO157 WT and *betTIBA-otsBA* mutant in leaf wounds on live lettuce plants. Total EcO157 population size per single wound per leaf (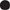) and percentage of the *betTIBA-otsBA* mutant in the total EcO157 population (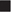) after their co-inoculation during wounding of leaves on whole potted lettuce plants. All values represent mean determinations from five (immediately after inoculation) and ten (during incubation) leaf wound disks sampled from random leaves and random plants. ^∗^, ^∗∗^, and ^∗∗∗^ indicate significant difference in the % of mutant over total EcO157 cells at 24, 48, and 72 h compared with the initial % at the time of inoculation (0 h), as per Dunnett’s multiple comparison test on transformed % data, with *P* < 0.05, 0.01, and 0.005, respectively. Error bars, standard error of the mean.

The competitive fitness of the mutant followed a different trend in wounds on leaves of whole lettuce plants since its proportion decreased throughout the entire incubation to reach 40.8% over 72 h, which was significantly different than that of 52.0% at the time of inoculation [arcsin(squareroot %)-transformed data; ANOVA, *P* < 0.01; Dunnett’s multiple comparison test, *P* < 0.005] (**Figure 8**). Application of exogenous GB to the wounds by its addition to the inoculum suspension did not appear to affect these competition dynamics (data not shown).

## Discussion

Plant injury allows for breaching of the natural physical barrier to infection and offers new opportunities for colonization by members of its microbiota, as is common in eukaryotic organisms at large. Human enteric pathogens have emerged as the causal agents of numerous outbreaks of foodborne illness associated with fruit and vegetables. These pathogens have the ability to exploit mechanically damaged or diseased tissue to enhance their growth and survival on plants (Aruscavage et al., 2006, 2008; Brandl, 2008; Goudeau et al., 2013; Simko et al., 2015), which are otherwise not as hospitable to enteric pathogens as the intestinal milieu (Brandl, 2006; Wiedemann et al., 2015). Epidemiological trends showing that a large proportion of outbreaks of EcO157 infection linked to leafy vegetables is associated with minimally processed product (Mandrell, 2009), which inherently harbors wounded tissue, indeed point to an important role of plant lesions in this emergence.

Bacterial colonists may benefit from increased nutrient availability in wounded plant tissue, but also must contend with the physico-chemical stresses resulting from plant cell leakage and the plant innate defense response to injury, as we demonstrated previously (Kyle et al., 2010). A close examination of the EcO157 transcriptome in lettuce lysates in the latter study, and in MAP shredded lettuce in a recent RNAseq-based study (unpublished data) revealed that the *betTIBA* genes for the import of choline and its catabolism to GB were upregulated, indicating that the human pathogen responds to low water potential in wounded lettuce leaves by production of this osmoprotective compound.

Studies by Beattie and co-workers revealed that the plant colonist *P. syringae* experiences water limitation during colonization of the leaf apoplast and responds by *de novo* synthesis of the compatible solutes trehalose and *N*-acetylglutaminylglutamine amide, and by uptake of quaternary ammonium compounds, including choline (Wright and Beattie, 2004; Chen et al., 2013; Yu et al., 2013). Free choline, choline-containing compounds, and GB have been measured in various amounts in the leaf tissue of Iceberg and romaine lettuce, cabbage, and spinach (Zeisel et al., 2003). Additionally, evidence for the extracellular presence of choline compounds and their availability to *P. syringae* in bean leaves and germinating seeds was obtained using whole-cell bacterial reporters (Chen et al., 2013). Using LC-MS analysis, we demonstrate here that (1) choline, but not GB, nor proline, is present in large amounts in Iceberg lettuce leaves; and (2) during growth in Iceberg leaf lysates with enhanced osmotic strength, EcO157 TW14588 WT strain accumulated high intracellular levels of GB and was more depleted in choline than its *betTIBA* mutant. In contrast, the *betTIBA* mutant contained little GB but accumulated choline in high concentration. Additionally, the *betTIBA* mutant was significantly impaired in growth compared with the WT in lettuce lysate under low water potential conditions. Hence, choline is sufficiently abundant in lettuce tissue contents to be imported and used via the BetTIBA pathway in EcO157 for osmoprotection in that environment. We observed also that in contrast to pseudomonads (Chen et al., 2013; Wargo, 2013b), EcO157 does not utilize choline and GB as substrates for growth, thus making these solutes highly available to regulate intracellular osmotic potential. Despite the upregulation of *betTIBA* in lettuce lysate and the indication that EcO157 uses this pathway to produce GB to respond to osmotic stress, the similar growth of the *betTIBA* mutant and WT strains in lettuce homogenates with 550 mM NaCl (Student’s *t*-test, *P* > 0.05) suggests that the mutant may have relied also on other compatible solutes under these conditions. Indeed, transcriptomic studies by Kocharunchitt et al. (2012, 2014) revealed that several osmoprotective pathways other than the Bet pathway could be induced in EcO157 Sakai by low water activity in complex culture medium that, similarly to lettuce lysate, may have provided a range of osmoprotective compounds.

Further evidence of the role of the BetTIBA pathway in the fitness of EcO157 in injured plant tissue was obtained by monitoring population dynamics after single inoculations of the WT and *betTIBA* mutant onto cut/shredded lettuce leaves under MAP conditions, a commodity with which outbreaks of EcO157 infections linked to produce have been predominantly associated (Mandrell, 2009). The population sizes of the *betTIBA* mutant were significantly lower than those of the WT after 1 and 6 h of colonization of MAP Iceberg lettuce (Student’s *t*-test, *P* < 0.05), indicating that first, a natural choline pool is available to EcO157 in injured lettuce tissue and second, deficiency in choline import and its conversion to GB impacts the ability of EcO157 to tolerate osmotic stress on cut leaves and fully exploit that habitat.

Close examination of the fate of choline leaked from injured lettuce cells using tracing experiments and LC-MS analysis revealed that the supplementation of the EcO157 inoculum suspension with D9-choline resulted in the presence of D9-GB in the WT cells 6 h after colonization of MAP lettuce whereas this compound was present in the *betTIBA* mutant cells only at levels near the detection limit of the system. Uninoculated lettuce leaves to which D9-choline was added in the same experimental set up did not yield any D9-GB above the detection limit, corroborating the results of our LC-MS analysis of lettuce homogenates and that of others (Zeisel et al., 2003), which showed that lettuce is not a high GB-producing plant species. This also indicated that the indigenous microflora on lettuce produced little, if any, GB in cut lettuce under MAP conditions.

Consistent with previous reports that trehalose must be synthesized *de novo* for trehalose-mediated osmoprotection in *E. coli* (Klein et al., 1991), addition of this solute to minimal medium amended with high NaCl concentrations did not rescue growth of the WT, nor that of the single *otsBA* and *betTIBA* mutants (data not shown). In our previous transcriptomic studies, *otsBA* was downregulated in EcO157 in leaf lysates (Kyle et al., 2010) and cut lettuce leaves (unpublished), consistent with the high activity of the GB biosynthetic pathway in the pathogen in these two environments and the previously reported post-translational repression of OtsA by high intracellular levels of GB (Giaever et al., 1988). Indeed, in the absence of GB as a compatible solute in minimal medium with high salt, the *betTIBA* mutant grew similarly to the WT in our study, presumably because a lack of GB allowed for the activity of the trehalose biosynthetic pathway in the two strains. In order to avoid such compensation in osmoprotection by *de novo* trehalose synthesis in the *betTIBA* mutant, we also deleted the *otsBA* operon in the EcO157 Δ*betTIBA* background. This deletion of *otsBA* caused complete growth inhibition of the resulting Δ*betTIBA*-Δ*otsBA* double mutant compared with the WT in minimal medium amended with high NaCl concentrations. Moreover, transformation of the single *otsBA* mutant or double *betTIBA-otsBA* mutant with complementation plasmid pMBotsBA enabled greater growth of the complemented mutants than of the WT, further emphasizing the effectiveness of *de novo* produced trehalose in osmoadaptation of EcO157.

Competitive fitness studies in MAP cut lettuce and leaf lesions of whole live plants revealed that the *betTIBA-otsBA* mutant had significantly less adaptability in the injured plant tissue than the parental strain. In both study systems, the representation of the double mutant in the total EcO157 population decreased following inoculation at a ratio of 1:1 with the WT strain, indicating that the human pathogen coped more effectively with the conditions experienced in wounded leaf tissue when capable of mounting a full osmotic stress response. Similarly, a *P. syringae* mutant defective in BetT and two other major transporters of compatible solutes displayed reduced epiphytic fitness on bean and soybean leaves (Chen et al., 2013). In our study, the competitive fitness of the *betTIBA-otsBA* mutant showed a constant decline over 72 h after co-inoculation in the leaf lesions of whole lettuce plants. This is in contrast to MAP cut lettuce, in which the mutant had rebounded by 24 h post-inoculation, as indicated by the increase of the percentage of the mutant in the total EcO157 population. This increase in competitive fitness followed an adaptation phase (characterized by only a slight population size increase of 0.7-log within 6 h after inoculation). The period of recovery in competitive fitness of the mutant after initial adjustment to the environmental conditions of cut leaf tissue under MAP conditions correlated with the period when the MAP bag atmosphere was near depletion in O_2_ content. It is possible that by this time, the *betTIBA* mutant had already shifted its strategy to other osmoprotective mechanisms due to a lack of BetTIBA activity and thus was better adapted to colonizing the cut tissue, whereas the WT strain needed to readjust its osmoresponse once *betTIBA* expression was inhibited by these low levels of O_2_ (Lamark et al., 1996) after 6 h of incubation. A lack of choline availability in the leaked contents of MAP lettuce was unlikely to have caused this change in population dynamics between the mutant and the WT since ample choline was still available to both strains even after 24 h of incubation.

Despite its significant proportional loss in the total EcO157 population in injured lettuce leaf tissue, the mutant retained considerable overall fitness. This may be expected considering the redundancy in osmotic stress response pathways in *E. coli* (Kempf and Bremer, 1998) and the range of compatible solutes that may be present in lettuce cell contents. Additionally, the heterogeneity in environmental conditions in damaged lettuce would also imply that not every single EcO157 encountered osmotic stress or mounted the same physiological response. More specifically in our study system, heterogeneous conditions experienced by EcO157 cells would first result at a larger scale from the presence of both intact and injured tissues. At the microscale, environmental heterogeneity as experienced by bacterial colonists in the phyllosphere has been observed in numerous studies (Joyner and Lindow, 2000; Brandl et al., 2001; Miller et al., 2001; Axtell and Beattie, 2002; Remus-Emsermann et al., 2012; Parangan-Smith and Lindow, 2013; Ryffel et al., 2016). Heterogeneity in water availability as sensed by individual *P. syringae* cells has been demonstrated on intact bean leaf surfaces using whole cell reporters (Axtell and Beattie, 2002). In damaged leaf tissue, heterogeneity may prevail at the microscale due to variations in water potential and in choline abundance driven by differences in the extent of injury among plant cells and hence, cell leakage across microsites colonized by the human pathogen, as well as due to variations in O_2_-regulated *betTIBA* expression. Additionally, levels of free choline in damaged tissue are modulated by the plant defense response to injury, which involves rapid reincorporation of choline into plant membrane phospholipid phosphatidyl choline for cell repair in order to avoid water stress (Tasseva et al., 2004). This choline sink may have contributed to the overall decrease in choline concentration that we measured overtime in washes of cut lettuce and of leaf lesions of whole plants.

Consistent with EcO157 cellular accumulation of GB and not choline by the WT in lettuce lysate, amendment of hypertonic M9 medium with GB enhanced the growth rate of the WT more effectively than amendment with choline. This suggested that unlike in *P. syringae* (Chen and Beattie, 2008), GB uptake, which proceeds in *E. coli* via the ProU and ProP systems, or OmpC porins (Kempf and Bremer, 1998), is more efficient than choline import and its conversion to GB (in the presence of BetTIBA) under high osmolality. It is noteworthy, however, that chemical analyses of lettuce leaf contents showed that GB was present at low levels and that the related osmoprotectant proline betaine was undetectable (de Zwart et al., 2003; Zeisel et al., 2003). It will be valuable to determine if GB import would be a preferred osmoprotective strategy in EcO157 when GB is available in high abundance such as in the tissue of spinach leaves, which contain unusually high levels of this compound compared with other plant species (de Zwart et al., 2003; Zeisel et al., 2003). EcO157 contamination of minimally processed baby spinach caused a large outbreak in the United States in 2006 (Wendel et al., 2009), and differences in the ability of EcO157 to colonize damaged leaves of spinach and romaine lettuce have been reported (Khalil and Frank, 2010).

Given that leaf injury is inherent to agricultural pre-harvest practices and fresh-cut processing of leafy vegetables, it is critical to fully understand the physiology and behavior of enteric pathogens in this important ecological niche that provides a nutritional environment highly favorable to their proliferation. Our study demonstrates that the human pathogen EcO157 rapidly experiences osmotic stress in mechanical lesions of lettuce leaves and draws from an abundant pool of plant-derived choline to synthesize GB as a compatible solute for osmoadaptation. This opportune use of choline by EcO157 while other preferred osmoprotectants, such as proline, proline betaine and GB, are naturally scarce in lettuce tissue, illustrates the remarkable adaptability of enteric pathogens to a range of physicochemical conditions and stresses on plants.

## Author Contributions

MB, RS, and RT designed experiments. RS, LH, YZ, and MB performed experiments. RS and MB analyzed data and wrote the manuscript. RT and LH edited the manuscript.

## Conflict of Interest Statement

The authors declare that the research was conducted in the absence of any commercial or financial relationships that could be construed as a potential conflict of interest.

## Footnotes

1 http://www.graphpad.com
