# Supplementary material for: Escherichia coli O157:H7 Converts Plant-Derived Choline to Glycine Betaine for Osmoprotection during Pre- and Post-harvest Colonization of Injured Lettuce Leaves
Source: Front Microbiol. 2017 Dec 8;8:2436. doi: 10.3389/fmicb.2017.02436 (PMC5727454; doi:10.3389/fmicb.2017.02436)
Supplement: Supplementary file 1 [file Data_Sheet_1.pdf]

Table S1. Primers used in this study

Utility	primer name	Sequence (5'-3')	Reference
Site-directed mutagenesis			
	betT_pKD3-FWD	ATGATTCCCGCGCCATATGCAGACATGGCGCGGTTTTATGCAATAACAAGTGTAGGCTGGAGCTGCTTC	
	betA_pKD3-REV	CTGTCCCCATACCTGGAACGGTAACTCTCAATCTGATCGGTTCCTGAGATGGGAATTAGCCATGGTCC	
	otsB_pKD4-FWD	AGCGCGTTCTGCGCAACACAATAAGAAAAGAGAAGGAGGAGAACCGGGTGGTGTAGGCTGGAGCTGCTTC	
	otsA_pKD4-REV	GTGAGGTCGATGTGCTGTTAGTTCCACTTACGGGAGATTAACCGCTCCTAATGGGAATTAGCCATGGTCC	
Confirmation			
	betT_F	CAAAAGCAGGAGAAATACATTTAATATAC	
	betA_R	GTCACTATGAAGCATGAGAGTTAC	
	otsB_F	TGTTATCTCCGCCTGCGTTT	
	otsB_R	AAAAGCGGCCATTTTCCACC	
	otsA_F	GGCTATGACTGGGCACAACA	
	otsA_R	TGCCTACGGTGAGTTAAGCG	
Plasmid Construction			
	otsSpeI-F	ACTGACACTAGTTCGCACCAAAATTATTATCTTTGTTCTCTCTGGC,	
	otsSacI-R	ACTGACGAGCTCCTACACAAGCTTAGGAAAGGTAGCAACTTTATCG	
	BetSpeI-F	ACTGACACTAGTTCACGCGTCCGGGAACATC	
	BetSacI-R	ACTGACGAGCTCTCAGTTCAAATCACGACTCATTTTTTTCGCTCTTAC	

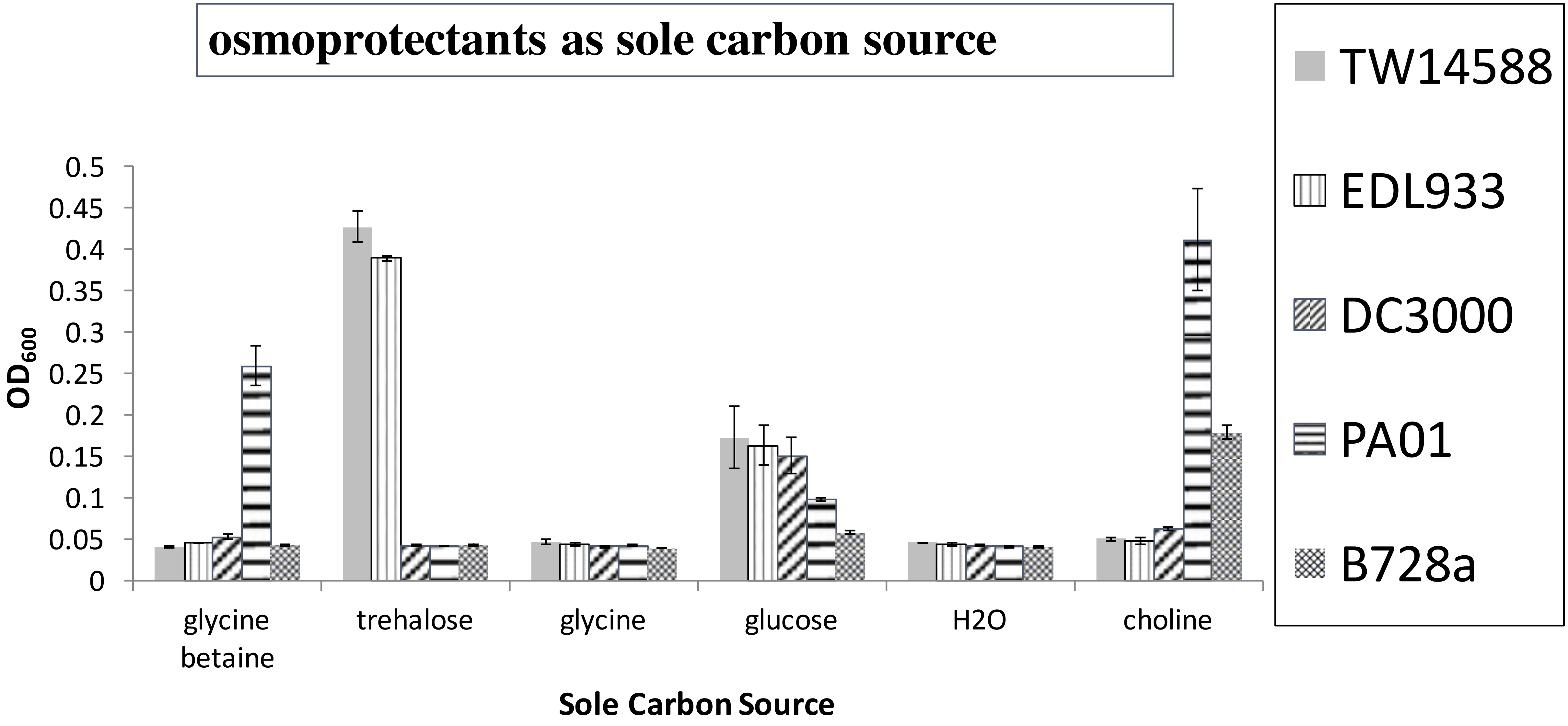

Figure S1. The osmoprotectants choline and glycine betaine do not serve as sole carbon sources for *E. c*oli O157:H7 TW14588 and EDL933 in M9 minimal medium. Trehalose is a growth substrate but periplasmic breakdown during import was reported to prevent its use as an exogenously supplied osmoprotectant during osmotic stress in *E. coli* K12 (Klein, Ehmann, and Boos, 1991). *Pseudomonas syringae* B728a and *P. aeruginosa* PAO served as positive control strains for osmoprotectant consumption, while *Pseudomonas syringae* pv. tomato DC3000 served as an additional control. Growth was measured as OD_{600} of culture after 48 h @ 28ºC. Glycine, glycine betaine, and choline were provided at a final concentration of 30 mM. Trehalose and Glucose were provided at a final concentration of 5 mM.

Reference:

Klein, W., Ehmann, U., and Boos, W. (1991). The repression of trehalose transport and metabolism in *Escherichia coli* by high osmolarity is mediated by trehalose-6-phosphate phosphatase. *Res. Microbiol.* 142, 359–371.
